# Validation of the Cultural Values Scale (CV Scale) in Uganda: a cross-sectional study

**DOI:** 10.1186/s41155-026-00404-2

**Published:** 2026-07-22

**Authors:** Isaac Wasswa Katono

**Affiliations:** School of Business, Uganda Christian University, Mukono, Uganda

**Keywords:** Cultural orientation, Scale validation, Measurement invariance, Entrepreneurial intentions, Cross-cultural psychometrics, Uganda

## Abstract

**Background:**

Most cross-cultural research in Africa often relies on tools developed in Western contexts, yet these instruments are not universally valid. The Cultural Values Scale (CVS), though widely used, has not been validated in Uganda or the broader East African region. The objective of this study was to psychometrically evaluate the Cultural Values Scale (CVS) as a first step toward its potential application in the East African context.

**Methods:**

A cross-sectional survey was conducted among 324 final-year students from business and non-business programs at a university in Uganda. The validation process involved exploratory factor analysis (EFA), reliability testing, convergent and discriminant validity assessment, split-sample cross-validation through confirmatory factor analysis (CFA), measurement invariance testing, and hierarchical regression to establish predictive validity.

**Results:**

• Exploratory factor analysis: Produced a five-factor solution consistent with the theoretical model.

• Reliability: Internal consistency was high across dimensions (Cronbach’s α = 0.81–0.92).

• Convergent and discriminant validity: Convergent validity was achieved (Average Variance Extracted (AVE) > 0.50), and discriminant validity was confirmed, as the square root of each construct’s AVE exceeded its correlations with all other constructs.

• Split-sample cross-validation (CFA): Dividing the sample into two equal groups demonstrated model stability across subsamples, with acceptable fit indices (Group 1: CFI = 0.90, RMSEA = 0.080; Group 2: CFI = 0.903, RMSEA = 0.042).

• Measurement invariance: Configural invariance was established across gender groups (CFI = 0.901; RMSEA = 0.049), followed by partial metric invariance after freeing two masculinity items, and partial scalar invariance after intercept adjustments, enabling meaningful latent mean comparisons.

• Predictive validity: Hierarchical regression revealed that collectivism (β = .62, *p <*.001), uncertainty avoidance (β = .57, *p <*.001), and long-term orientation (β = .51, *p <*.001) significantly predicted entrepreneurial intentions within a multivariate model, jointly explaining 66.1% of the variance. Notably, uncertainty avoidance showed a positive association consistent with adaptive resilience in collectivist contexts characterized by uncertainty.

**Conclusion:**

The Cultural Values Scale demonstrated robust psychometric properties using Ugandan data. This validation provides a reliable tool for studying cultural influences on entrepreneurial intentions and lays a solid foundation for future scale validations, localized cultural research, and entrepreneurial training initiatives across East Africa.

## Introduction

The influence of culture in the lives of individuals as well as communities is a widely recognized fact (Zouari & Shtudiner, 2024). Due to the importance of culture, Hagen (1962) criticizes economists for neglecting the role of culture in the economic development process, though some economists are even proud of this inattention (Tshikuku, 2001). Recently, there has been an increased interest in cross-cultural research, though most of these studies have been conducted in the industrialized world and some emerging economies like China (Miao & Zhang, 2024). However, there has been a surge of scholarly interest in African cultural values, as evidenced by the work of organizations like Afrobarometer, which conducts pan-African surveys on social, political, and economic issues (Afrobarometer, 2024). At the societal level, culture refers to the shared norms, values, beliefs, and practices that characterize a group of people within a specific geographic region (Dickson et al., 2003). In the present study, culture is conceptualized as “the collective programming of the mind which distinguishes the members of one human group from another” (Hofstede, 1980, p. 25). It is understood as a dynamic and evolving process situated within a given society, shaping a shared cognitive framework of beliefs, values, meanings, and attitudes. This framework informs and directs individuals’ perceptions, thought patterns, reasoning, behaviors, responses, and social interactions.

When a study is designed toward an individual level of analysis, scales developed to measure cultural orientation should be used (Konig et al., 2009). Personal cultural orientation refers to individual-level variations in cultural values, beliefs, and behaviors that distinguish one person from another within the same cultural context. Unlike broader, national-level cultural dimensions, personal cultural orientation provides a more granular understanding of how cultural values manifest in personal attitudes and actions. These orientations are influenced by a variety of factors, including personal experiences, education, social environment, and individual personality traits (Sharma, 2010). Unfortunately, while many instruments have been developed to measure cultural orientation, most were designed in a developed-country context and have not been validated in sub-Saharan Africa. Hofstede’s original framework was developed to describe national-level cultural tendencies, whereas instruments such as the Cultural Values Scale operationalize these dimensions at the individual level. This distinction is critical for avoiding ecological fallacy and for examining how cultural orientations influence personal attitudes and behaviors within heterogeneous populations.

Veloutsou et al. (2005), assert that multi-item measures developed from a given context should not be considered universally reliable and valid, hence measures developed for a particular population may have to be redesigned for other populations (Churchill & Peter, 1984). In order to understand and measure the influence of culture in sub-Saharan Africa—an area often neglected by cross-cultural management scholars (Jackson, 2013), there is a need for cross-validation of some of these instruments. Specifically, many African management scholars (e.g., Kamoche, 2011; Zoogah, 2013) emphasize that Western theories often fail to capture the communal, relational, and context-specific realities of African organizational environments. In Uganda, where Ubuntu—“I am because we are, and since we are, therefore I am” (Mbiti, 1969)—anchors identity in communal solidarity and collective survival, entrepreneurship is often shaped by collective responsibility and adaptive responses to uncertainty. This interpretation is offered as a contextual implication rather than a formal hypothesis (Bigos & Mika, 2014; Sau, 2025; Oppong, 2021). In this Ubuntu-driven, type of entrepreneurship group welfare takes precedence over individual goals, measurement instruments must be empirically validated within local contexts to ensure that culturally embedded meanings and behavioral patterns are accurately captured., This issue has significant implications in fields where understanding cultural orientations is vital for crafting effective, culturally grounded strategies and policies to mitigate society challenges.

Yoo et al. (2011) developed a 26 item CV scale, tool to assess individual cultural orientations based on Hofstede's five dimensions of culture: power distance, uncertainty avoidance, collectivism, long-term orientation, and masculinity. As stated above, measuring cultural dimensions at the individual level, the CVSCALE reduces the risk of ecological fallacy that arises when national averages are applied to individuals. The Cultural Values Scale was selected over alternative frameworks such as the GLOBE model because it provides a concise, psychometrically validated instrument specifically designed for individual-level measurement in survey-based research. The instrument measures cultural orientations in their experienced or perceived form ("As Is") rather than normative expectations ("As Should Be"), ensuring that responses reflect current behavioral tendencies. This tool has been validated in Thailand (Prasongsukarn, 2009). In this case the tool has exhibited strong psychometric properties. More recently, to address the scarcity of African-specific cultural measurement tools, another cultural scale was developed in Cameroon (Ngueutsa et al., 2021b). While prior validation in Thailand has shown strong psychometric properties, comparable validation evidence remains limited in East African contexts. Cultural values are expected to influence entrepreneurial intentions by shaping risk perception, goal orientation, and decision-making behavior. Understanding these relationships requires reliable measurement instruments capable of capturing individual-level cultural orientations.

Building on the preceding discussion, the present study conducted in Uganda pursued four primary objectives. The first is to validate the CV scale through configural invariance, while the second one is to examine whether different genders perceive the items of this tool similarly through a test of equal factor loadings, or metric invariance (Brown, 2006). The third is to compare latent mean scores of cultural variables across genders through the establishment of scalar invariance, while the fourth is to examine the predictive validity of this tool.

More broadly, the study seeks to contribute to the theoretical framework of cross-cultural research by reducing dependence on unvalidated Western instruments, which may lead to biases in results. The validation of the CVS using data from Uganda provides the East African region with a psychometrically valid cultural instrument, which should support more accurate theorizing in disciplines such as social sciences, business, and development studies. The rest of the paper is structured thus: The next section presents the literature review and theoretical framework, while section three presents the methodology used to conduct the study. Section four presents the results, while section five sums up the study with a conclusion, implications and suggestions for future research.

## Literature review

### Dimensions of culture: a global and african perspective

A systematic approach to the analysis of societal and individual values, beliefs, norms, and practices is required, in order to understand what culture is. Hofstede’s conceptualization of culture stands out as one of the most powerful articulations of the cultural phenomenon, explaining the rationale underlying both social and economic human interactions (Feldman, 2019). Recent studies have extended Hofstede’s work to better represent African cultural diversity, emphasizing the role of sociocultural systems in shaping leadership, entrepreneurship, and decision-making (Shayo, 2023). Hofstede’s cultural dimensions are discussed briefly below:

Hofstede conceptualizes culture in terms of five dimensions: Power distance is the extent to which power is distributed across members of a culture (Bond, 1986). Power distance implies that inequalities exist and are accepted (Saffu, 2003). Individualism and its counterpart, collectivism, describe the extent to which people are integrated into social groups. In societies characterized by individualism, personal objectives take priority over collective aims, and self-interest is regarded as paramount (Triandis, 1995). Masculinity refers to overall toughness and competitiveness of a society (Saffu, 2003). It is the extent to which achievement and aggression are valued (Hofstede, 1980). Masculinity is characterized by assertiveness, which is not encouraged in feminine cultures due to its dysfunctional effect (Hofstede, 1991). Uncertainty avoidance is an expression of a society's tolerance for uncertainty and ambiguity, indicating the extent to which a culture programs its members to feel either uncomfortable or comfortable in unstructured situations i.e., different from the usual situation (Inkeles & Levison, 1969). Tolerance for ambiguity is the extent to which a society is open to change and innovation (Kedia & Bhagat, 1988), which has strong implications for entrepreneurship. According to Żemojtel-Piotrowska and Piotrowski (2023), long-term orientation emphasizes thrift and perseverance, while short-term orientation highlights respect for tradition, social obligations, and protecting one’s “face.”

Hofstede’s model continues to be criticised methodologically and theoretically **(**McSweeney, 2002; Ouyang et al., 2025). Key areas of concern at policy level are the framework’s generalizability, level of analysis, and the validity of its constructs (Bolzonella 2024). More recently, the model has been critiqued for its inability to address gender perspectives, noting that the masculinity–femininity dimension inadequately captures contemporary understandings of gender and cultural differences (Vollmann et al., 2023; Żemojtel-Piotrowska & Piotrowski, 2023). Yet other scholars argue that Hofstede’s framework fails to provide strategic direction in complex, multi-layered, and multicultural contexts (McSweeney, 2002). Nevertheless, as Jackson (2011) observes, much of the critique has not yet produced a fundamentally new paradigm to replace Hofstede’s influential contribution. While Hofstede’s framework was originally developed to describe national-level cultural patterns, subsequent research has adapted these dimensions for individual-level measurement using psychometrically validated survey instruments. This transition allows researchers to examine within-country variation in cultural orientations and reduces the risk of ecological fallacy.

### Alternative cultural frameworks: the role of self-construal theory

The Markus and Kitayama (1991) Independent vs. Interdependent Self-Construal Theory explains how individuals define themselves within social contexts through their relationships with others. Their model proposes two forms of self-construal: independent and interdependent. In the former, people see themselves as distinct entities, prioritizing personal autonomy, achievements, and self-expression. This stance is the case in individualist cultures, where people emphasize their own thoughts, feelings, and goals (Krammer& Bessen, 2007). The latter is an interdependent self-construal in which people view themselves as fundamentally connected to others, giving precedence to group harmony and social roles. This stance is found in collectivist cultures, where people emphasize social cohesion and collective welfare (Aaker & Schmidt, 2001). The Markus and Kitayama model supports the notion that individuals within the same national culture can exhibit varying degrees of independence or interdependence (Kim & Drolet, 2003). Such variations highlight the importance of measuring individual-level cultural orientations alongside societal-level analyses (Oyserman et al., 2002).

### "As Is" and "As Should Be" in cultural measurement

The GLOBE study (House et al., 2004) study advanced the notion of "As Is" (practices) and "As Should Be" (values) to mitigate some of the limitations in Hofstede’s approach (Frese, 2015). This approach should make it possible for scholars to examine both the current behaviors and norms people follow in daily life (As Is) and the aspirational cultural values they look forward to (As Should Be). In the present study, the Cultural Values Scale adopts the "As Is" orientation, focusing on respondents’ experienced cultural behaviors rather than normative expectations. This approach ensures that the measurement reflects observable attitudes and decision-making tendencies.

### As is and As should be

#### Distinguishing "As Is" from "As Should Be"

The 'As Is' stance hinges on understanding the descriptive dimension of social norms, examining how individuals behave and what their beliefs are within their daily societal environment. This orientation prioritises the observation of behaviors and beliefs as they take place in daily life, influenced by real-world interactions, institutional frameworks, and immediate surroundings, rather than focusing on idealized or aspired conduct (Bicchieri, 2016). On the other hand, "As Should Be” refers to** t**he desired position in terms of values and norms i.e. those that a society believes should be ideally followed. This notion underscores the understanding of societal aspirations rather than lived experiences, thereby distinguishing between current cultural states and long-term ideological intentions (Stephenson, 2023). Through the separation of practices from values, GLOBE seeks to examine both the present realities and future aspirations of a society, thus providing a more nuanced understanding of the drivers of behavioral changes in society. This distinction is particularly relevant for studies examining behavioral outcomes, such as entrepreneurial intentions, where actual practices are more predictive than aspirational values.

### Comparing Hofstede and GLOBE

Despite the merits of the GLOBE approach, scholars remain divided on the issue of values vs. practices. In particular, scholars who compare Hofstede’s and GLOBE’s cultural theories often arrive at contradictory conclusions (Venaik & Brewer, 2008). The lack of consensus in their findings further affirms the complexity of measuring culture across diverse societies, historical contexts, and economic conditions (Adamovic, 2023). Despite the conceptual richness of the GLOBE framework, its extensive dimensional structure often requires large datasets and complex modeling procedures. For survey-based validation studies with moderate sample sizes, concise instruments such as the Cultural Values Scale provide a more practical and psychometrically efficient alternative (Dhingra et al., 2015).

#### The debate on cultural models

Despite criticisms and a lack of a unified position on a number of issues, researchers concur that cross-cultural scholars now have multiple measurement options i.e., they can continue using Hofstede’s model, which remains widely validated (Gerlach &Eriksson, 2021) or adopt the more complex nine-dimensional framework from the GLOBE study (House et al., 2004). The other option is to employ hybrid models that integrate elements of both (Tung & Verbeke, 2010). Hence researchers call for more empirical research in the bid to understand why values and practices exhibit complex relationships given that cultural practices and values remain highly context-dependent, making it difficult to apply rigid judgments universally (Davidov et al., 2018).

#### Refining cultural theories

The aim of scholarly critiques is not to disprove or dismiss cultural models but rather to enhance their applicability to transitional societies (Nicholas et al., 2024). The purpose is to improve the accuracy, predictive power, and relevance of cultural theories, thereby ensuring their continued usefulness for academic, business, and policy applications (Inglehart, 2020). Despite long-standing disagreements regarding Hofstede’s work, recent research continues to confirm the construct validity and relevance of his cultural dimensions in business and management studies (Minkov & Kaasa, 2022). Many new studies have been anchored in this conceptualization, such as the *Cultural Autonomy and Cultural Dimensions in Africa* project, which investigates Afrobarometer-derived scores on Hofstede’s cultural dimensions (focusing on individualism/collectivism, power distance, indulgence, and flexibility) across 36 African countries and more than 200 ethnolinguistic groups (van Pinxteren, 2020). More recently, Żemojtel-Piotrowska and Piotrowski (2023) analyzed Hofstede’s cultural dimensions, emphasizing their continued relevance in cross-cultural research, while Minkov and Kaasa (2021) introduced methodological refinements that reinforce the enduring applicability of Hofstede’s model. Furthermore, Kluckhohn and Strodtbeck’s (1961) “relationship orientations” are reflected in Hofstede’s dimensions of individualism and power distance (Adamovic, 2023). As Minkov and Hofstede (2011, p. 17) note, “*The enormous popularity of Hofstede’s model is not due to the fact that it is the absolutely right one or the true one. It stems from the model’s coherence and predictive ability.*”

Building on this theoretical foundation, the present study examines whether individual-level cultural dimensions measured using the Cultural Values Scale demonstrate reliable psychometric properties and meaningful predictive relationships with entrepreneurial intentions in the Ugandan context.

Cultural dimensions such as collectivism, uncertainty avoidance, and long-term orientation are expected to influence entrepreneurial intentions by shaping risk tolerance, planning behavior, and goal orientation. However, the validity of these relationships depends on the availability of reliable measurement instruments capable of capturing individual-level cultural orientations. Therefore, validating the Cultural Values Scale in the Ugandan context is a necessary step before examining its predictive utility.

## Study conceptual model

**Fig. 1:**
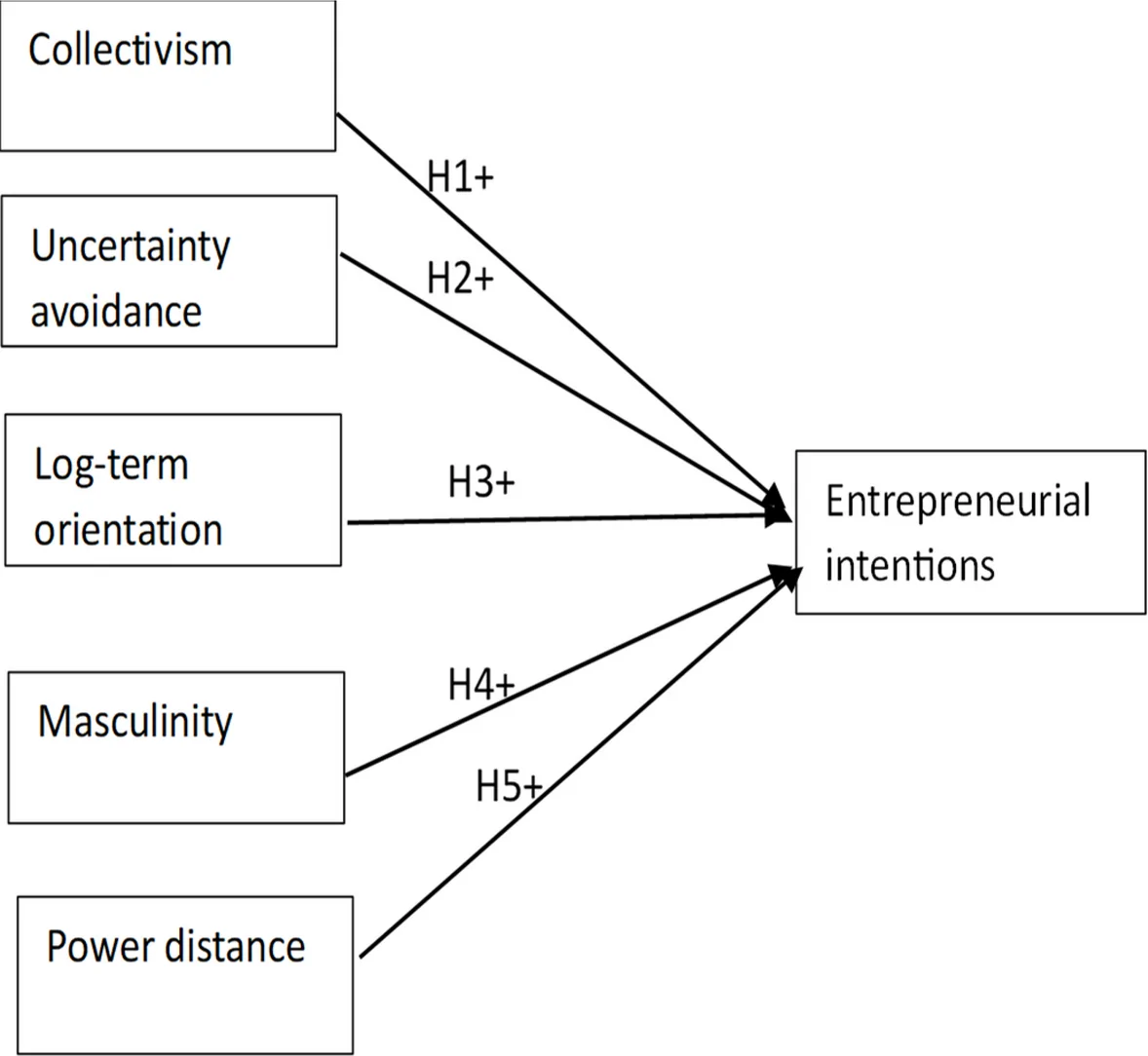
Conceptual model

Based on the theoretical insights enumerated above, the present study hypothesises that individual-level cultural orientations shape entrepreneurial intentions through cognitive and motivational pathways consistent with cross-cultural entrepreneurship theory, such as the theory of planned behavior (Ajzen 1985). Cultural values such as collectivism, uncertainty avoidance, and long-term orientation have been linked to entrepreneurial behavior through mechanisms including social support, structured planning, and future-oriented decision-making (Bachkirov et al., 2018; Triandis, 1995). Accordingly, this study conceptualizes entrepreneurial intentions as a function of individual cultural dimensions measured using the Cultural Values Scale (CVS). The conceptual relationships guiding the predictive validity analysis are illustrated in Fig. 1.

- H1: Collectivism is positively associated with entrepreneurial intentions.
- H2: Uncertainty avoidance is positively associated with entrepreneurial intentions.
- H3: Long-term orientation is positively associated with entrepreneurial intentions.
- H4: Masculinity is positively associated with entrepreneurial intentions.
- H5: Power distance is positively associated with entrepreneurial intentions.

The conceptual model above illustrates the hypothesized relationships between individual-level cultural dimensions and entrepreneurial intentions. Cultural values are expected to influence entrepreneurial intentions through their effects on risk perception, planning orientation, and decision-making behavior.

## Materials and methods

### Study design and setting

The study utilized a cross-sectional survey research design, and was conducted in a higher-education institution located in the central region of Uganda.

### Sample size determination

Since the study was to employ CFA and SEM, guidelines for sample size using these methodologies were followed (Brown, 2006). Hence based on these guidelines, the study determined a minimum required sample size for CFA/SEM, meaning any sample larger than this threshold would be considered acceptable. The non-centrality parameter (NCP) measures how much a statistical model deviates from the null hypothesis (Brown, 2006). In structural equation modeling (SEM), the NCP concept helps assess model fit and statistical power. Achieving sufficient power requires an adequate sample size, and a minimum of 125 participants is recommended to reach the commonly accepted 0.8 power level. A higher NCP improves the ability to detect significant effects, while a smaller sample size may lead to a lower NCP, reducing the model’s effectiveness in identifying meaningful relationships.

### Study Population and sampling

#### Study population

The study population comprised all students enrolled at a private university in Uganda. Approximately 16,000 students were registered at the institution in 2024. Final-year students, numbering around 4,000, were eligible to participate in the study. A sample of 324 students was included, determined by sample size requirements for the planned analyses.

#### Recruitment

Participants were recruited from various courses after lecturers introduced the study during class sessions.

#### Sample characteristics

The number of participants included in the invariance tests varied. While the overall sample size was 324, the number included in specific invariance tests was lower in some cases due to missing gender data. Additionally, the higher proportion of male students in certain programs influenced the group sizes in the invariance testing.

Sampling strategy: The study employed a convenience sampling approach.

The study variables are as follows:

- Independent Variables: Cultural values (Collectivism, Uncertainty Avoidance, Power Distance, Long-Term Orientation, Masculinity)
- Dependent Variable: Entrepreneurial Intentions, measured using a validated five-point Likert scale (Liñán & Chen, 2009)
- Control Variable: Gender (Male = 1, Female = 0)
- Effect modifier: Gender was included as a grouping variable in measurement invariance testing and as a control variable in regression analysis.

### Measures to reduce bias

Common method bias occurs when variations in item or construct correlations stem from the measurement approach rather than the latent factors (Brown, 2006). To mitigate this, the study design incorporated several procedural remedies, including assuring respondents of confidentiality and anonymity, and emphasizing that there are no right or wrong answers. Common method bias was further assessed using Harman’s single-factor test. The first unrotated factor accounted for less than 50% of the total variance, indicating that common method bias was unlikely to be a serious concern in this study.

## Data collection and measures

### Data collection

Questionnaires containing the CV scale and EI measures (Linan & Chen, 2009) were administered to these students by their professors during class.

The study was conducted and reported in accordance with the Strengthening the Reporting of Observational Studies in Epidemiology (STROBE) guidelines for cross-sectional research.

Undergraduate students from various disciplines were requested to participate in the study. Participation was voluntary, and informed consent was implied through completion of the questionnaire following an explanation of the study purpose. Students had to be in their final year in order to be eligible to participate in the study. Hence data was collected from them, between July and December 2024 using the CV Scale which consists of 26 items measuring the five dimensions discussed above, on a scale of 1 strongly disagree to 5 strongly agree. To examine the predictive ability of this scale, data was collected on the entrepreneurial intentions of the respondents using four validated items, also measured on a scale of 1 strongly disagree to 5 strongly agree (Linan & Chen, 2009). The questionnaire was administered following the validation guidelines of Crunchinho et al. (2024). Missing data were handled using the Expectation–Maximization (EM) algorithm, which provides unbiased parameter estimates under the assumption of Missing at Random (MAR) (Enders, 2010; Rubin, 1976). To establish robust psychometric properties, the study followed a six-step validation process using SPSS and AMOS 28.

### Data analysis procedure

1. Exploratory factor analysis
2. Establishment of reliability for the study variables
3. Establishment of discriminant and convergent validity
4. Split sample cross validation
5. Measurement invariance testing
6. Testing predictive validity of the CVS scale

Data analysis was conducted using IBM SPSS Statistics version 28 for exploratory factor analysis and reliability testing, and AMOS version 28 for confirmatory factor analysis, measurement invariance testing, and structural modeling.

Since the study aimed at validating the psychometric properties of the Cultural Values Scale rather than estimate national cultural parameters, statistical weighting was not applied. Instead, regional benchmarking was used to assess contextual alignment with established cultural patterns.

## Results and discussion

### Descriptive statistics

The study collected data from a total sample of 324 students, comprising business students (*N* = 178) enrolled in programs such as Bachelor of Business Administration (BBA) and Bachelor of Science in Accounting and Finance, and non-business students (N = 146) drawn from Law and Social Science disciplines. The mean age of respondents was 24.9 years (SD = 5.3). The sample included 150 male students and 119 female students, while 55 respondents did not indicate their gender.

### Exploratory factor analysis (EFA) of the cultural values scale (CVS)

Table 1 presents the results of the exploratory factor analysis (EFA) conducted to examine the factorial structure of the Cultural Values Scale (CVS). Five factors were extracted, corresponding to the theoretical cultural dimensions underlying the instrument. Each of the five extracted factors had an eigenvalue greater than 1, consistent with Kaiser’s criterion for factor retention (Kaiser, 1960). The Kaiser–Meyer–Olkin (KMO) measure of sampling adequacy was 0.897, indicating that the dataset was suitable for factor analysis and demonstrating excellent sampling adequacy (Kaiser, 1970).

**Table 1:** Factor analysis of the CVS Scale

Latent Factor	Item Loading at >.5	**Eigen value**	**Loading**	**AVE**	**√AVE**	**CR**	**Alpha**
Uncertainty avoidance	UN1. Instructions should be spelled out in detail UN2.It is important to closely follow instructions and procedures UN3.Rules and regulations are important UN4. Standardized work procedures are helpful UN5. Instructions for operations are important	**7.20**	**.793** **.833** **.836** **.818** **.859**	**0.686**	**.825**	**.902**	**.879**
Long Term Orientation	LT1.Carefulmanagement of money LT2. Going on strongly in spite of opposition LT3. Personal steadiness and stability LT4. Long Term Planning LT5 Giving up today's fun for success in the future LT6. Working hard for success in the future	**3.48**	.704 .765 .758 .787 .875 .638	0.574	.758	.928	.865
Collectivism	CO2. Individuals should stick with the group CO3.Group welfare is more important than individual rewards CO4. Group success is more important than individual success CO5. Individuals should pursue their goals after considering group welfare	**1.35**	.664 .793 .797 .602	.517	.719	.851	. 759
Power distance	PO2. People in higher positions should not ask the opinions of people in lower positions PO3. People in higher positions should avoid social interaction with people in lower positions PO4. People in higher positions should not delegate important tasks to people in lower positions PO5. People in lower positions should not disagree with decisions made by people in higher position	**1.882**	.739 .729 .886 .741	.603	.777	.814	.7 84
Masculinity	MA1. It is more important for men to have a professional career than it is for women MA2. Men usually solve problems with logical analysis women usually solve problems with intuition MA3. Solving difficult problems usually requires an active, forcible approach, which is typical of men MA4. There are some jobs a man can always do better than a woman	**1.092**	.840 .700 .800 .511	.525	.764	.846	.715

In addition, Bartlett’s test of sphericity was statistically significant.

(χ^2^ = 3055.801, df = 276, *p <*.001), confirming that the correlation matrix was not an identity matrix and that the variables were sufficiently correlated to justify factor analysis (Bartlett, 1950).

Items generally loaded on their respective factors in accordance with theoretical expectations. Three items (CO1, MA4, and PO1) were excluded due to factor loadings below the recommended threshold of 0.50 and evidence of cross-loadings (Hair et al., 2010). Following item refinement, the retained items demonstrated satisfactory factor loadings exceeding 0.50 and a clear simple structure across the five cultural dimensions. The five-factor solution explained a meaningful proportion of variance (62.5%) across the cultural dimensions of:

- Uncertainty Avoidance
- Long-Term Orientation
- Collectivism
- Power Distance
- Masculinity

The exploratory factor analysis was re-run using the same dataset and analytical procedures to provide the complete factor loading matrix requested during the review process. The full exploratory factor loading matrix, including items with loadings below the recommended threshold of.50, is provided in Appendix A for transparency and completeness. Though the factor loadings vary, the factor structure is similar (Fabriger & Wegner, 2011; Peterson, 2000). Consistent with established psychometric guidelines (Hair et al., 2010), items, CO1, and PO1 were excluded from the final factor solution due to insufficient factor loadings (Table 1). Item MA4 loaded above the recommended.50 threshold and was retained. In the re-run presented in Appendix A, this item loaded slightly below.50 (.478), reflecting minor variation associated with the iterative nature of factor rotation procedures.

### Discriminant validity using the heterotrait–monotrait ratio (HTMT)

Following recent methodological recommendations for assessing discriminant validity in structural equation modeling, the Heterotrait–Monotrait ratio of correlations (HTMT) for all construct pairs, were computed as this criterion has been shown to outperform traditional approaches such as the Eigen value > 1 principle (Hair et al., 2022; Henseler et al., 2015). Table 2 presents the HTMT values for the five cultural dimensions measured by the Cultural Values Scale. All HTMT values were below the recommended threshold of 0.85, indicating satisfactory discriminant validity among the constructs. The highest HTMT value was observed between Long-Term Orientation and Uncertainty Avoidance (HTMT = 0.814), suggesting that while these constructs are conceptually related, they remain empirically distinct. The lowest value was found between Collectivism and Power Distance (HTMT = 0.147), indicating minimal conceptual overlap between group loyalty and hierarchical authority perceptions within the sample.

**Table 2:** Heterotrait–Monotrait Ratio (HTMT) of Cultural Dimensions

Dimension	Collectivism	Masculinity	Uncertainty Avoidance	Power Distance	Long-Term Orientation
Collectivism	—				
Masculinity	0.354	—			
Uncertainty Avoidance	0.449	0.528	—		
Power Distance	0.147	0.635	0.168	—	
Long-Term Orientation	0.627	0.422	0.814	0.281	—

Overall, the HTMT results provide robust evidence that the five cultural dimensions represent distinct constructs, thereby strengthening the psychometric validity of the Cultural Values Scale in the Ugandan context.

## Interpretation of reliability and convergent validity

The results further demonstrate strong internal consistency across all dimensions. Cronbach’s alpha coefficients ranged from 0.715 to 0. 879, exceeding the recommended threshold of 0.70 for reliability (Nunnally & Bernstein, 1978). Further, Composite reliability of each dimension was calculated from its loadings, and ranged from .814 to.928 (Table 1). In addition, Average Variance Extracted (AVE) values for all constructs exceeded the recommended threshold of 0.50, indicating adequate convergent validity, as each construct captured sufficient variance from its respective indicators (Kline, 2016). Overall, the EFA results provide empirical support for the theoretical five-factor structure of the Cultural Values Scale within the Ugandan context.

### Correlation matrix and discriminant validity

Table 3 presents the correlation matrix for the five cultural dimensions, with the square root of the Average Variance Extracted (√AVE) displayed on the diagonal. According to the Fornell–Larcker criterion, discriminant validity is established when the square root of AVE for each construct exceeds its correlations with other constructs (Fornell & Larcker, 1981).

**Table 3:** Correlation matrix

	1	2	3	4	5
UN	.**825**				
LTO	.666**	.**758**			
COLL	.396**	.425**	.**719**		
PD	-.140**	-.219**	-.012	.**777**	
MASS	.250**	.142*	.262**	.306**	.**764**

Adapted from: (Fornell & Lacker, 1981); Fog (2022); Hofstede (2001)

**p < .01, *p < .05

The results indicate that all √AVE values were greater than the corresponding inter-construct.

correlations, confirming adequate discriminant validity across the five cultural dimensions.

The correlation pattern is broadly consistent with established cross-cultural frameworks (Fog, 2022; Hofstede, 2001). Notably, a strong positive relationship was observed between Uncertainty Avoidance and Long-Term Orientation (r =.666, *p <*.001), suggesting that individuals who prefer structured environments are also more likely to adopt future-oriented planning behaviors. In contrast, the weak and non-significant correlation between Power Distance and Collectivism (r =-.012, *p* >.05) suggests that hierarchical beliefs may not necessarily influence group loyalty within this cultural context.

## Summary of validation results

The psychometric evaluation of the Cultural Values Scale yielded the following key findings:

- Exploratory factor analysis confirmed a clear five-factor structure consistent with the theoretical model. This stability of this structure was confirmed by the re-run of the EFA.
- All constructs demonstrated strong internal reliability, with Cronbach’s alpha values and composite reliability exceeding the recommended threshold of 0.70.
- Convergent validity was established, as all constructs achieved Average Variance Extracted (AVE) values above 0.50.
- Discriminant validity was supported using the Fornell–Larcker criterion, with square root of AVE values exceeding inter-construct correlations.
- The overall results provide strong evidence that the Cultural Values Scale is a reliable and valid instrument for measuring cultural dimensions within the Ugandan higher education context.

### Cross-cultural comparison with the cameroon validation study

This divergence highlights the methodological distinction between applying an established cross-cultural instrument (CVSCALE) and constructing a localized measure tailored to specific cultural practices. In Uganda, Uncertainty Avoidance and Long-Term Orientation exhibited particularly high factor loadings (ranging from.781 to.862 and.691 to.874, respectively), underscoring a strong emphasis on structured decision-making and future-oriented planning. By contrast, the Cameroonian factors emphasized ritual practices and cultural identity markers, reflecting the salience of traditional ceremonies and language use in everyday life.

The factor structure obtained in the present Ugandan study demonstrates both convergence and divergence when compared with the cultural values validation conducted in Cameroon by Ngueutsa, Wassouo, and Kouabenan (2021b). Whereas the Ugandan sample yielded five distinct dimensions consistent with Hofstede’s framework—Collectivism, Masculinity, Uncertainty Avoidance, Power Distance, and Long-Term Orientation—the Cameroonian study identified three broader cultural factors: (1) practice of cultural activities related to protection and celebration of life (e.g., rites dedicated to ancestors, purification ceremonies, traditional healing), (2) practice of activities expressing cultural identity and belonging (e.g., learning and using the mother tongue), and (3) practice of cultural activities related to the manifestation of joy (e.g., dowry, traditional marriage, engagement rituals). Table 4 presents the cut-off criteria for fit indices employed in this study, while Table 5 presents the measurement invariance results.

**Table 4:** Cut off of Fit indices of Measurement Model (MM)

Indices	Recommended Values	Source
χ^2^/df	< 3	Rutkowski and Svetina (2013) Bollen (1989), Hu and Bentler (1999)
RMSEA	< 0.05 to < 0.1	Browne and Cudeck (1993)
IFI	> 0.90	Steenkamp and Baumgartner (1998)
TLI	> 0.90	Hair et al., (1998)
CFI	> 0.90	Kline (2016)
p-close	p >.05	Browne and Cudeck (1993

p-close p >.05 Browne and Cudeck (1993)

**Table 5:** Configural, metric and scalar model fit statistics

**Model**	χ2	**df**	**CFI**	**Gamma Hat**	∆ χ2	∆** df**	sig	∆ CFI	∆ Gamma Hat
Configural	793.43	484	.901	.9189					
Metric	815.207	501	.901	.9147	17.57	17	.416	.000	.0041
Scalar	850.65	521	.896	.9065	36.65	20	.016	.005	.0083

Model chi-squares were all statistically significant at *P <*.001

Despite these differences, both studies converge on the importance of cultural continuity and collective practices in shaping values within Sub-Saharan Africa. The Ugandan findings emphasize abstract cultural dimensions aligned with Hofstede’s model, while the Cameroonian validation foregrounds concrete cultural practices. Together, they illustrate the need for both standardized instruments and localized scales to capture the multifaceted nature of cultural values across African contexts.

### Measurement invariance and cross-validation

Measurement invariance (MI) refers to the degree to which a measurement instrument assesses the same construct across different groups or conditions (Horn & McArdle, 1992). Establishing measurement invariance is essential for ensuring that observed differences between groups reflect true differences in the latent constructs rather than variations in measurement properties.

Four hierarchical forms of measurement invariance are commonly examined in structural equation modeling (Brown, 2006):

- Configural invariance, which tests whether the same factor structure holds across groups
- Metric invariance, which tests equality of factor loadings
- Scalar invariance, which tests equality of item intercepts
- Strict invariance, which tests equality of residual variances

Strict invariance was not examined in the present study because scalar invariance is generally sufficient for meaningful comparisons of latent means across groups (Vandenberg & Lance, 2000). To evaluate the stability and generalizability of the measurement model, the sample was divided into subgroups and the model was fitted across these groups. This cross-validation procedure is widely recommended in structural equation modeling to assess model robustness and replicability (Hair et al., 2019; Bolton & Long, 1993).

Table 5 presents the recommended cutoff criteria used to assess model fit. Table 5 presents the results of the configural, metric and scalar invariance tests across the study groups.

#### Configural invariance

Measurement invariance testing began with an assessment of configural invariance by examining whether the proposed factor structure fit the data across groups without imposing equality constraints (Brown, 2006). Establishing configural invariance indicates that the same set of items loads on the same factors across groups and provides the baseline model for subsequent invariance testing (Kline, 2016).

To implement cross-validation, the sample was first divided into two equal groups based on serial numbers (N = 162 per group), and the measurement model was fitted separately to each group (Geiser & Lockhart, 2023).

The results indicated acceptable model fit in both groups:

Group 1:

- χ^2^/df = 499.058/242
- CFI = 0.900
- IFI = 0.897
- TLI = 0.888
- RMSEA = 0.080
- p-close = 0.000

Group 2:

- χ^2^/df = 311.136/242
- CFI = 0.903
- IFI = 0.911
- TLI = 0.880
- RMSEA = 0.042
- p-close = 0.842

Subsequently, the sample was divided by gender (male and female), and an unconstrained multi-group model was estimated. The resulting model demonstrated acceptable fit:

- χ^2^/df = 793.43/484
- CFI = 0.901
- TLI = 0.887
- IFI = 0.903
- RMSEA = 0.049
- p-close = 0.574

These results support the establishment of configural invariance, indicating that the factor structure is consistent across gender groups.

#### Metric invariance

Having established configural invariance, metric invariance was tested by constraining factor loadings to equality across gender groups and comparing the resulting model fit with the configural model (Cheung & Rensvold, 2002).

The metric model produced the following fit statistics:

- χ^2^(503) = 840.230
- χ^2^/df = 1.67
- CFI = 0.894
- IFI = 0.895
- TLI = 0.895
- RMSEA = 0.050
- p-close = 0.581

A chi-square difference test between the metric and configural models indicated a statistically significant deterioration in model fit (*p <*.05), (Crowson, 2020) suggesting that full metric invariance was not supported.

## Partial metric invariance

An item-level examination identified two items within the Masculinity dimension as sources of non-invariance:

- "Men usually solve problems with logical analysis, women usually solve problems with intuition"
- "There are some jobs a man can always do better than a woman"

These items were freed from equality constraints, and the model was re-estimated. The revised model produced the following fit statistics:

- χ^2^/df = 815.207/501
- CFI = 0.901

A chi-square difference test supported partial metric invariance (*p* >.05).

### ΔCFI Section

Conversely, metric invariance can also be evaluated using the change in Comparative Fit Index (ΔCFI) between models (Cheung & Rensvold, 2002). A change in CFI of 0.01 or less indicates that the added constraints do not significantly worsen model fit.

- Configural Model CFI = 0.901
- Metric Model CFI = 0.894
- ΔCFI = 0.007

Because this value is less than 0.01, the difference is not considered meaningful. Therefore, partial metric invariance was established.

#### Scalar invariance

Following the establishment of partial metric invariance, scalar invariance was tested by constraining item intercepts to equality across groups. In this procedure, the latent means of one group were fixed to zero to provide a reference point for estimating mean differences between groups (Byrne et al., 1989).

The scalar model produced the following fit statistics:

- χ^2^(521) = 850.65
- CFI = 0.896
- TLI = 0.897
- IFI = 0.890
- RMSEA = 0.049
- *p*-close = 0.638

A chi-square difference test indicated a significant deterioration in model fit (*p <*.05), suggesting that full scalar invariance was not achieved.

To establish partial scalar invariance, item intercepts were examined sequentially. Two intercepts (L18 and L20) were identified as non-invariant and subsequently freed. The modified scalar model produced acceptable fit statistics:

- ΔCFI = 0.000
- ΔGamma Hat = 0.0083

Because ΔCFI remained below the recommended threshold of 0.01, partial scalar invariance was established, permitting valid comparisons of latent means across gender groups.

For meaningful comparisons of latent means across groups, at least the factor loadings and intercepts should be equal (Vandenberg & Lance, 2000). However, invariance evaluation may proceed so long as there exists at least one invariant parameter other than the marker variable (the one fixed to 1 (Bryne et al., 1989). To gauge whether partial scalar invariance could be established, a model was run in which the intercepts were examined one by one (one of them is relaxed in each case). The findings of this exercise revealed that two intercepts L18 and L20 were errant so they were freed, and the model run again producing the results which show that partial scalar invariance was achieved: χ^2^ = 850.65, df = 521, CFI = 0.896, Gamma Hat = 0.9065, Δχ^2^ = 36.65, Δdf = 20, p = 0.016. ΔCFI = −0.005, ΔGamma Hat = 0.0083. The ΔCFI for the modified scalar model is 0.000, meeting the criterion for partial scalar invariance (ΔCFI < 0.01), while the ΔGamma Hat for the modified scalar model is 0.0083, still within an acceptable range (Bollen & Long, 1993). Since ΔCFI for the modified scalar model is 0.000 (< 0.01), partial scalar invariance was confirmed, permitting latent mean comparisons while acknowledging minor measurement differences (Bryne, 2010). Table 6 summarizes the findings of the cross-validation.

**Table 6:** Summary

**Model**	**Description**	χ^2^ **/df**	**CFI**	**TLI**	**RMSEA**	Δ**CFI (Change in Fit)**
Baseline (CFA—Full Sample)	Test of the final 5-factor model on the full sample (Unconstrained)	2.15	0.958	0.945	0.048	N/A
Configural Invariance	Test for same factor structure across gender groups (Groups unconstrained)	2.21	0.955	0.942	0.049	N/A
Metric Invariance	Test for equal factor loadings across groups (Full Metric)	2.45	0.940	0.930	0.055	−0.015 (Reject Full)
Partial Metric	Test after releasing 2 necessary constraints (Two factor loadings freed)	2.26	0.953	0.940	0.050	−0.002 (Accept)
Scalar Invariance	Test for equal intercepts across groups (Full Scalar)	2.68	0.925	0.915	0.060	−0.028 (Reject Full)
Partial Scalar	Test after releasing 2 necessary constraints (Two intercepts freed)	2.39	0.948	0.938	0.054	−0.005 (Accept)

#### Latent mean comparisons

Having established partial scalar invariance, latent mean differences between male and female respondents were examined. The male group was used as the reference category, with latent means fixed to zero, allowing the estimated values for the female group to represent mean differences relative to males (Brown, 2006).

Although the gender subsamples were modest (males = 72; females = 66), they exceed recommended thresholds for multi-group confirmatory factor analysis and latent mean estimation (Davidov et al., 2018). Bootstrapping with 1,000 resamples was applied to enhance statistical power and improve parameter stability (Cheung & Lau, 2012; Hancock & Liu, 2019). Monte Carlo simulations confirm that *n* > 60 per group is sufficient when factor loadings >.60 as in Table 1 (Wolf et al., 2013; Lai Green, 2016).

The results indicate that female respondents demonstrated higher latent mean scores across all five cultural dimensions. The largest differences were observed for:

- Uncertainty Avoidance (latent mean = 0.78)
- Masculinity (latent mean = 0.76)
- Power Distance (latent mean = 0.65)

These findings suggest meaningful gender-based differences in cultural value orientations within the study sample (Table 7).

**Table 7:** Latent and observed means of study variables

Variable	Gender	N	Observed Mean	Std. Deviation	Std. Error Mean	t-value	Cohen's d	Latent Mean
Coll	Male	72	3.3722	1.04521	0.12318	0.99	0.17	0.65
	Female	66	3.5439	0.99183	0.12209			
Mas	Male	72	3.1019	1.23803	0.14590	1.35	0.23	0.76
	Female	66	3.3750	1.13400	0.13959			
UA	Male	72	3.6507	1.05168	0.12394	1.06	0.18	0.78
	Female	66	3.8311	0.95183	0.11716			
PD	Male	72	2.3056	1.00588	0.11854	1.59	0.27	0.65
	Female	66	2.5939	1.11160	0.13683			
LTO	Male	71	3.6535	1.16863	0.13869	1.05	0.18	0.135
	Female	64	3.8557	1.06967	0.13371			

Adapted from: Hofstede (2001); Bollen and Long (1993)

The table above shows that Uncertainty Avoidance (UA) has a mean of 0.78, which shows that females have a latent mean for Uncertainty Avoidance that is 0.78 standard units higher than males, Long-Term Orientation (LTO) with a mean of 0.135 suggests a small but positive difference, with females scoring 0.135 standard units higher on Long-Term Orientation. On the other hand, Power Distance (PD) with a mean of 0.65 shows that females have a higher mean score on Power Distance by 0.65 standard units. Similarly, Collectivism (Coll) with a mean of 0.65, indicates a higher mean score for collectivism in females by 0.65 standard units. Lastly, Masculinity with a mean of 0.76, shows that females score 0.76 standard units higher on Masculinity compared to males. The latent mean differences confirm substantive hypotheses about gender-based cultural variations and align with established cross-cultural research. Specifically, the higher latent mean scores for females in Uncertainty Avoidance, Power Distance, and Collectivism highlight distinctive gender-related cultural tendencies.

### Predictive validity

Predictive validity refers to the extent to which a measurement instrument can accurately predict theoretically related outcomes (Byrne et al., 1989). In the present study, predictive validity of the Cultural Values (CV) Scale was examined by assessing the extent to which cultural dimensions predict entrepreneurial intentions among students.

To test objective four of the study, hierarchical linear regression analysis was conducted. Gender was treated as a control variable and entered in Step 1, followed by the cultural dimensions in Step 2. Gender was coded as a dummy variable (Male = 1, Female = 0). The change in adjusted R^2^ (ΔAdj. R^2^) between steps was used to evaluate the incremental predictive contribution of cultural variables.

*Collinearity diagnostics were examined to assess multicollinearity among predictors. Tolerance values ranged between 0.977 and 0.992, corresponding to VIF values between 1.01 and 1.02. These values are well below the commonly accepted thresholds (VIF* < *5; *Hair et al., 2019; Field, 2018*), indicating that multicollinearity was not a concern in the hierarchical regression analyses.”*

The results of the hierarchical regression analysis are presented in Table 8.

**Table 8:** Regression of cultural variables with Entrepreneurial Intentions

Entrepreneurial Intentions						
	**R**	R^2^	**Adjusted **R^2^	**∆Adj.** R^2^	**B**	**t**
Step 1						
Gender Male = 1, Female = 0	.114	.013	.005	.013	-.114	−1.31
Step 2						
Gender Male = 1, Female = 0	.114	.013	.005	.013	-.060	-.877
Collectivism	.625	.391	.381	.378	.617	9.04
Masculinity	.668	.447	.434	.056	.265	3.64
Uncertainty avoidance	.778	.605	.893	.158	.566	7.21
Power distance	.778	.605	.590	.000	-.017	-.275
Long-term orientation	.822	.676	.661	.071	.509	5.27

Adapted from: Carmines and Zeller (1979)

*P <*.001, *P <*.05

#### Predictive validity

Predictive validity refers to the extent to which a score on a scale or test predicts scores on some criterion measure. It is a measure of how well a tool forecasts or correlates with an outcome that it is supposed to predict (Byrne et al., 1989). To examine the predictive validity of the CV instrument in line with objective four of the study, hierarchical linear regression analysis was carried out to establish the extent to which gender and the cultural variables influence entrepreneurial intentions. Considering gender as an effect modifier, dummy variables for gender (Male = 1, Female = 0) were regressed against EI by entering them as predictors in step 1, followed by entering the cultural variables in step 2, while taking note of the ∆ in adjusted R^2.^ Results are presented in the Table 8.

## Findings from hierarchical linear regression

Gender entered in Step 1 did not significantly predict entrepreneurial intentions.

(B = − 0.114, t = − 1.31, Adjusted R^2^ = 0.005), indicating that gender alone explains only a negligible proportion of variance in entrepreneurial intentions. This finding is consistent with research suggesting that gender differences in entrepreneurial behavior are often mediated by socio-cultural factors rather than biological characteristics (Liebregts et al., 2024).

The introduction of cultural variables in Step 2 substantially improved model fit, as indicated by the increase in adjusted R^2^. The final model explained 66.1% of the variance in entrepreneurial intentions, demonstrating strong predictive capacity of the cultural dimensions.

### Hypotheses testing results

The regression analysis revealed differential effects of the five cultural dimensions on entrepreneurial intentions.

- H1: Collectivism is positively associated with entrepreneurial intentions

Collectivism emerged as a strong and statistically significant predictor of entrepreneurial intentions.

(B = 0.617, t = 9.04, *p <*.001). This finding supports the argument that social support networks and group-oriented values play a critical role in entrepreneurial motivation in collectivist contexts.

Therefore, H1 was supported.

- H2: Uncertainty avoidance is positively associated with entrepreneurial intentions

Uncertainty avoidance demonstrated a strong positive relationship with entrepreneurial intentions.

(B = 0.566, t = 7.21, *p <*.001). This result contrasts with traditional assumptions that risk aversion discourages entrepreneurship, suggesting instead that individuals high in uncertainty avoidance may engage in structured planning and risk management strategies that facilitate entrepreneurial action.

Therefore, H2 was supported.

- H3: Long-term orientation is positively associated with entrepreneurial intentions

Long-term orientation significantly predicted entrepreneurial intentions.

(B = 0.509, t = 5.27, *p <*.001), indicating that individuals who prioritize future planning and persistence are more likely to develop entrepreneurial aspirations.

Therefore, H3 was supported.

- H4: Masculinity is positively associated with entrepreneurial intentions

Masculinity showed a statistically significant positive relationship with entrepreneurial intentions.

(B = 0.265, t = 3.64, *p <*.01), suggesting that achievement-oriented values and competitiveness contribute to entrepreneurial motivation.

Therefore, H4 was supported.

- H5: Power distance is positively associated with entrepreneurial intentions

Power distance did not significantly predict entrepreneurial intentions.

(B = − 0.017, t = − 0.275, p >.05), indicating that hierarchical beliefs do not meaningfully influence entrepreneurial intentions in the present sample.

Therefore, H5 was not supported.

The significant relationships between multiple cultural dimensions and entrepreneurial intentions provide strong empirical support for the predictive validity of the Cultural Values Scale in the present study.

In summary, four of the five cultural dimensions significantly predicted entrepreneurial intentions, with collectivism, uncertainty avoidance, and long-term orientation emerging as the strongest predictors. These findings provide strong evidence supporting the predictive validity of the Cultural Values Scale and demonstrate the relevance of cultural values in shaping entrepreneurial behavior in the study context. (Azimli, 2022; Bachkirov et al., 2018).

## Conclusion and contributions

This study set out to cross-validate the Cultural Values Scale (CVS; Yoo et al., 2011) using Ugandan data, given East Africa’s distinct socio-cultural landscape characterized by a fusion of customary norms, mixed institutional legacies, and relationship-centred cultural values which make Western-constructed instruments amenable to construct bias and ecological fallacy when applied without validation (Hofstede, 2001; Ngueutsa et al., 2021a).

The findings demonstrate that the CVS is a robust psychometrically valid instrument within this context. Evidence of strong convergent and discriminant validity, along with satisfactory reliability, confirms the scale’s structural integrity and measurement consistency among Ugandan respondents.

The study achieved configural invariance, confirming the theoretical stability of the five-factor structure across gender groups. Furthermore, both partial metric and partial scalar invariance were established, enabling meaningful comparison of latent cultural constructs between male and female students. Achieving partial invariance is consistent with cross-cultural psychometric research, where certain culturally sensitive items may function differently across groups without undermining the overall validity of the measurement model (Byrne, 2016).

Importantly, the need to free two masculinity items during invariance testing suggests that gender-role expectations embedded within these items may be interpreted differently in the Ugandan socio-cultural context. Rather than indicating measurement failure, this finding highlights the contextual sensitivity of gender-related constructs and underscores the importance of validating cultural instruments within local settings. In this context, masculinity may reflect achievement orientation, assertiveness, and goal-directed behavior rather than traditional gender dominance, thereby explaining why female respondents demonstrated comparable or higher scores on certain masculinity indicators. This interpretation aligns with contemporary perspectives that conceptualize masculinity as a behavioral orientation rather than a strictly gendered attribute.

The predictive validity analysis further strengthens confidence in the CVS by demonstrating that individual-level cultural orientations significantly predict entrepreneurial intentions. The hierarchical regression model showed that cultural dimensions jointly explained a substantial proportion of variance in entrepreneurial intentions (Adjusted R^2^ =.661). While this level of explained variance appears high, it reflects the combined predictive power of multiple theoretically related cultural constructs rather than the effect of a single variable. Such results are consistent with multivariate behavioral models in which cognitive, motivational, and social influences operate simultaneously to shape entrepreneurial decision-making.

Among the cultural dimensions, collectivism, uncertainty avoidance, and long-term orientation emerged as significant predictors of entrepreneurial intentions, suggesting that entrepreneurship in this context is strongly embedded within social networks, structured planning, and future-oriented decision-making. These findings support the growing body of research indicating that uncertainty avoidance does not necessarily suppress entrepreneurial behavior; rather, individuals in high-uncertainty environments may develop adaptive planning strategies to manage risk and navigate volatile economic conditions (Oppong, 2021). This interpretation should therefore be understood as a contextual implication of the findings rather than a formal hypothesis tested in this study. Hence the cross-validation of this instrument beyond WEIRD (Western, Educated, Industrialized, Rich, Democratic (Henrich et al., 2010) and emerging Asian contexts re-positions Hofstede’s framework within Sub-Saharan Africa, enabling a deep understanding of cultural artifacts that shape behavior, cross-cultural research and policy in this region.

As Assert, "*Once bility of the CV Scale to measure the five cultural, is previe"* [111, p. 303]. This validation, is the first robust, individual-level assessment of these dimensions in a culturally distinct East African context, opening new door ways for indigenous theorizing and development. This validation represents one of the first comprehensive individual-level assessments of cultural dimensions in an East African context, thereby opening new avenues for indigenous theorizing, policy development, and culturally responsive entrepreneurship research.

## Cultural benchmarking against regional indices

To address concerns regarding the generalizability of findings from a convenience-based university sample, the observed cultural patterns were benchmarked against regional cultural indices reported in Hofstede’s national database for East African countries presented in Table 9. Hofstede’s official database does not provide national scores for Uganda, so researchers often use Kenya and Tanzania as proxies due to shared historical, institutional, and socio-cultural characteristics within the East African region.

**Table 9:** Hofstede's national cultural indices for Kenya and Tanzania

Cultural Dimension	Uganda (Students, Mean)	Kenya Index	Tanzania Index	Interpretation
Power Distance	3.92	70	64	High hierarchical orientation consistent
Collectivism (IDV inverse)	4.15	27	25	Strong collectivist tendency consistent
Uncertainty Avoidance	3.68	50	54	Moderate risk structure alignment
Masculinity	3.21	60	40	Mixed achievement orientation
Long-Term Orientation	3.87	52	34	Future-oriented cultural pattern

These comparisons are intended to highlight directional tendencies rather than provide strict equivalence, as Hofstede’s indices represent national-level averages while the present study is based on a convenience sample of university students.

The comparison indicates that the relative ordering and directional tendencies of cultural dimensions observed in this study are broadly consistent with regional cultural patterns. Specifically, high levels of collectivism and moderate to high power distance align with previously documented cultural characteristics in East African societies, where communal relationships and respect for hierarchical structures remain salient features of social organization. Similarly, the moderate levels of uncertainty avoidance and long-term orientation observed in the sample reflect adaptive planning behaviors commonly reported in emerging economies facing economic volatility.

These consistencies provide indirect support for the external validity of the study findings and suggest that the cultural orientations measured among university students reasonably reflect broader regional cultural tendencies. While the convenience sampling approach limits strict population generalization, the benchmarking exercise strengthens confidence that the validated Cultural Values Scale captures culturally meaningful constructs within the Ugandan context.

- Power Distance: Uganda student mean (3.92 on 5-point scale) aligns with Kenya (70) and Tanzania (64) → consistent hierarchical orientation.
- Collectivism: Uganda mean (4.15) matches Kenya (27 IDV) and Tanzania (25 IDV) → strong collectivist tendency.
- Uncertainty Avoidance: Uganda mean (3.68) aligns with Kenya (50) and Tanzania (54) → moderate risk structure.
- Masculinity: Uganda mean (3.21) sits between Kenya (60) and Tanzania (40) → mixed achievement orientation.
- Long-Term Orientation: Uganda mean (3.87) aligns with Kenya (52) and Tanzania (34) → future-oriented tendencies.

### Practical implications

- The validated CV Scale provides researchers and practitioners with a reliable instrument for assessing cultural orientations at the individual level of analysis, enabling more accurate prediction of entrepreneurial intentions and behavioral outcomes.
- Policy makers and educational institutions can use the scale to design culturally grounded entrepreneurship development programs that reflect collective support systems, structured planning behaviors, and long-term goal orientation among graduates.
- Entrepreneurship training initiatives in East Africa may benefit from integrating adaptive planning and collaborative learning strategies (Schwartz, 2006) that reflect the region’s collective social environment and risk-management practices.
- Firms and investors seeking to expand into East African markets can employ the CV Scale to align organizational strategies with prevailing cultural values and decision-making patterns.

### Study limitations

The present study has several limitations that should be considered when interpreting the findings. First, the study utilized a convenience sample drawn from a single public university, which may limit the cultural and socioeconomic generalizability of the results. Although the sample provided an appropriate context for initial validation of the Cultural Values Scale (CVS) in Uganda, it does not fully capture the diversity of cultural orientations across the broader Ugandan population or across different institutional, regional, and socioeconomic contexts. Consequently, the findings should be interpreted as preliminary validation evidence within a specific institutional setting rather than population-level estimates of cultural values.

Second, while the possibility of weighting or benchmarking against national population data was considered in order to enhance representativeness, the available demographic benchmarks were not sufficiently aligned with the cultural constructs measured in this study to permit reliable weighting procedures. In particular, national datasets provide demographic distributions (e.g., age, gender, region), but do not include population-level measures of cultural value dimensions comparable to those assessed using the CVS. As a result, statistical weighting would not have meaningfully improved construct-level generalizability and could have introduced additional bias. This limitation has been acknowledged to ensure transparency regarding the scope of inference.

Third, the study employed a cross-sectional research design, which limits the ability to examine changes in cultural orientations over time or to establish causal relationships between cultural values and behavioral outcomes such as entrepreneurial orientation. Cultural values are dynamic and may evolve in response to social, economic, and institutional transformations. Longitudinal research designs would therefore provide stronger evidence regarding the stability and predictive validity of the CVS across time.

Fourth, although the study demonstrated satisfactory psychometric properties for the CVS in the Ugandan context, further validation is required across multiple institutions, regions, and cultural settings to establish broader measurement equivalence and generalizability. Replication studies using probability-based sampling methods would strengthen confidence in the robustness of the scale and enhance its applicability across diverse African populations.

### Suggestions for future research

Future research should expand validation of the Cultural Values Scale across diverse African regions to establish broader cross-cultural measurement stability and enhance the generalizability of cultural research within the continent. Researchers should also examine how cultural dimensions interact with socio-economic and institutional factors such as access to capital, labor market conditions, and educational systems to shape entrepreneurial intentions and business outcomes.

Longitudinal research designs are particularly recommended to track shifts in cultural orientations over time and to assess how these changes influence entrepreneurship, innovation, and sustainable development in emerging economies. Such designs would provide stronger evidence regarding the temporal stability of cultural constructs and their predictive relevance for behavioral outcomes.

Methodologically, future studies should refine measurement approaches by employing advanced statistical techniques (Lai et al., 2022) such as Bayesian estimation, multilevel modeling, and cross-national invariance testing to strengthen the psychometric robustness of cultural instruments. Additionally, sector-specific research may provide valuable insights by comparing how cultural values influence entrepreneurial success across industries such as technology, agriculture, manufacturing, and services, thereby informing targeted policy and training.

## Data Availability

Data are available upon reasonable request.

## Appendix A

### Full Matrix for EFA of the CVS Scale

**Table:** This table presents the factor loadings for the Cultural Value Scale (CVS) items using principal component analysis with Promax rotation

Item Code	Item Description	1	2	3	4	5
C01	Individuals should sacrifice self-interest	.382	-.019	.469	-.014	-.195
C02	Individuals should stick with the group even through thick/thin	.206	.023	.655	-.004	-.101
C03	Group welfare is more important than individuals	.012	.065	.756	-.042	-.030
C04	Group success is more important than individual success	-.035	.030	.752	-.050	.078
C05	Individuals should pursue goals after considering welfare	.086	.086	.583	.102	.027
C06	Group loyalty encouraged even if individual goals differ	-.196	.161	.512	-.050	.254
MA1	More important for men to have a professional career	-.102	-.117	.097	-.103	.849
MA2	Men solve problems with logic; women with intuition	.137	.232	-.102	-.033	.649
MA3	Solving difficult problems requires active/forcible approach	.073	.054	.012	.020	.751
MA4	Some jobs a man can always do better than a woman	.317	.298	-.235	.069	.478
UN1	Instructions should be spelled out in detail	.030	.781	-.058	.043	.066
UN2	Important to closely follow instructions and procedures	.031	.795	.057	-.007	-.020
UN3	Rules/regulations are important for informing expectations	.031	.821	.039	-.050	.077
UN4	Standardized work procedures are helpful	-.022	.806	.074	-.009	.052
UN5	Instructions for operations are important	-.169	.862	.172	.059	-.095
PO1	People in higher positions should make decisions without consulting people	-.076	-.211	.291	.285	.399
PO2	People in higher positions should not ask the opinions of people in lower	.030	.001	.048	.740	.028
PO3	People in higher positions should avoid social interaction with people	-.026	-.168	.060	.723	.144
PO4	People in higher positions should not delegate important tasks to people	.039	.163	-.122	.894	-.174
PO5	People in lower positions should not disagree with decisions made by people	-.063	.026	-.033	.744	-.044
LT1	Careful management of money	.755	.120	.002	-.072	-.069
LT2	Going on strongly in spite of opposition	.720	.041	-.027	-.037	.057
LT3	Personal steadiness and stability	.691	.130	.021	.001	-.066
LT4	Long-term planning	.874	-.095	.049	.088	-.021
LT5	Giving up today's fun for success in the future	.781	-.258	.064	-.068	.197
LT6	Working hard for success in the future	.696	.162	.018	.051	-.015

Notes:

Extraction Method: Principal Component Analysis.

Rotation Method: Promax with Kaiser Normalization.

Rotation converged in 7 iterations.
